# Scaffold-free endocrine tissue engineering: role of islet organization and implications in type 1 diabetes

**DOI:** 10.1186/s12902-025-01919-y

**Published:** 2025-04-21

**Authors:** Tugba Bal

**Affiliations:** Department of Bioengineering, Faculty of Engineering and Natural Sciences, Uskudar University, Istanbul, 34662 Turkey

**Keywords:** Diabetes, Pseudoislet, Cell-cell interactions, Beta-cell models, Pancreas (patho)biology, Drug screening, Beta-cell replacement therapy

## Abstract

Type 1 diabetes (T1D) is a chronic hyperglycemia disorder emerging from beta-cell (insulin secreting cells of the pancreas) targeted autoimmunity. As the blood glucose levels significantly increase and the insulin secretion is gradually lost, the entire body suffers from the complications. Although various advances in the insulin analogs, blood glucose monitoring and insulin application practices have been achieved in the last few decades, a cure for the disease is not obtained. Alternatively, pancreas/islet transplantation is an attractive therapeutic approach based on the patient prognosis, yet this treatment is also limited mainly by donor shortage, life-long use of immunosuppressive drugs and risk of disease transmission. In research and clinics, such drawbacks are addressed by the endocrine tissue engineering of the pancreas. One arm of this engineering is scaffold-free models which often utilize highly developed cell-cell junctions, soluble factors and 3D arrangement of islets with the cellular heterogeneity to prepare the transplant formulations. In this review, taking T1D as a model autoimmune disease, techniques to produce so-called pseudoislets and their applications are studied in detail with the aim of understanding the role of mimicry and pointing out the promising efforts which can be translated from benchside to bedside to achieve exogenous insulin-free patient treatment. Likewise, these developments in the pseudoislet formation are tools for the research to elucidate underlying mechanisms in pancreas (patho)biology, as platforms to screen drugs and to introduce immunoisolation barrier-based hybrid strategies.

## Graphical Abstract

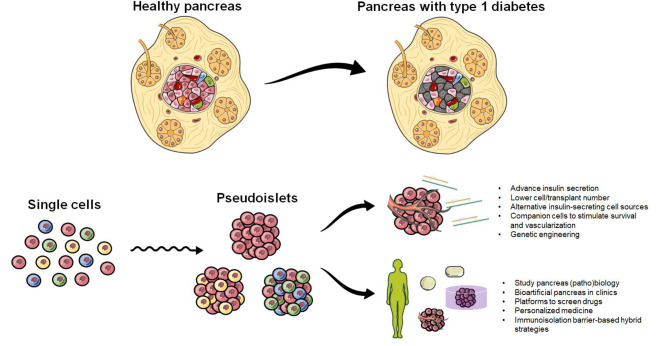

## Background

Pancreas is an organ in the abdominal cavity with a dual function through hormones to regulate certain aspects by the endocrine part and through the digestive pancreatic juice by the exocrine part [1]. Damage to this organ assisted by genetic factors under particular conditions is tied to or triggers health problems such as pancreatitis, neoplasms, pancreatic exocrine insufficiency, cystic fibrosis, Shwachman-Diamond Syndrome and diabetes associated with the exocrine and/or endocrine components [2]. Among this diverse disease set, diabetes is associated with chronic hyperglycemia which is the main consequence of lack of insulin (type 1 diabetes, T1D) or loss of the insulin production correlated with the insulin resistance (type 2 diabetes, T2D). As a complex disease originating from a dynamic interplay of causes contributed by distinct genetic and environmental factors, T1D is driven by the loss of non-self and self-discrimination for the beta-cells leading to an aggressive immune attack against these cells [3, 4].

In the genetic and environmental background of T1D pathogenesis, genetic criteria include human leukocyte antigen (HLA) and non-HLA loci. HLA genes are encoded in chromosome 6p21 and are grouped into class I and class II. Among these, especially, certain class II genes in HLA-DR and HLA-DQ have prominent roles to link T1D to the heredity [3]. For example, it has been demonstrated that HLA-DR3-DQ2 and/or HLA-DR4-DQ8 presence is the greatest risk factor, as the case study by Undlien et al. revealed that only 10% of the T1D patients did not have these haplotypes [5]. Additionally, the risk for islet autoimmunity highly correlates with siblings carrying HLA-DR3/4-DQ8 haplotype shared with proband siblings. The risk for these siblings was accounted for 85% by age 15 and lowered to 20% when the siblings did not share the same haplotype [6]. Patients carrying HLA-DR3-DQ2/DR3-DQ2 or DR3-DQ2/DR4-DQ8 genotypes were reported to have an increased risk of T1D and other diseases such as celiac disease concurrently [7]. Conversely, some of the HLA family members such as HLA-DR15-DQ6 were discovered as protective against T1D [8, 9], but cases with the failure of protection are reported [10]. Non-HLA genes associated with T1D involve in the immune system cell development and differentiation, as well as various signaling pathways with altered expression rates of soluble molecules or receptors such as insulin, protein tyrosine phosphatase non-receptor type 22 (PTPN22), cytotoxic T-lymphocyte antigen 4, interleukin (IL)-10 and interleukin 2 receptor subunit alpha. All these factors drive the loss of non-self and self-discrimination for the beta-cells leading to an aggressive immune attack [3]. Moreover, environmental factors such as gluten in the diet [11], obesity [12, 13], vitamin D deficiency [14, 15], infections including COVID-19 [16–19], gut microbiota [20, 21] and drugs [22, 23] are suggested to contribute to the pathophysiology of T1D.

After diagnosis, the main goal of the diabetes treatment is shaped around achieving normoglycemia. Due to destruction of the beta-cells, T1D treatment heavily depends on multiple daily combinatorial insulin injections [24–27]. In severe cases, beta-cell replacement therapy is applied where only pancreas/islet, simultaneous pancreas/islet and kidney or pancreas/islet after kidney transplantation is implemented into the treatment [24]. Among the pancreas and the islet transplantation, whole organ transplantation is the clinically applied strategy for the insulin independence for T1D patients whereas islet transplantation is a method which is still in clinical trials. However, donor source and eligibility criteria, surgical complications, contraindications to surgery (e.g. viral infections, arterial diseases, high body-mass index, alcohol usage, smoking) and life-long use of immunosuppressive drugs influence the distribution and the success of pancreas transplants [28, 29]. Alternatively, islet transplantation can be the solution to deal with some limitations of pancreas transplant. In this procedure, isolation of individual islets from the donor pancreas is required before transplantation to the patients. However, graft survival depends on the protection of the transplanted cells [30]. To address this bottleneck, an immunosuppressive regime is highly utilized in organ transplantations, yet this strategy of immunosuppression causes adverse effects on the transplanted cells as well as the body. To eliminate this, in 2000s, Edmonton protocol was established by Shapiro et al. that employs glucocorticoid-free immunosuppressive protocol of sirolimus, low-dose tacrolimus, and daclizumab (a monoclonal antibody against the IL-2 receptor) and, was put into the practice in clinical trials [31]. However, donor shortage and source, and related issues such as disease transmission [32], requirement of multiple transplants [33] and risks of transplantation [34] limit widespread application of the islet transplantation in the clinics.

To the clinics as well as to the research, artificial islets can be a possible choice to eliminate several of these drawbacks. Constructed with recent technologies, these systems are widely applied to the endocrine tissue research, especially as an intermediate between preclinic and clinics [35]. This review focuses on the most frequent scaffold-free design called “pseudoislet” and their current applications in T1D research. The ability of these 3D cell assemblies to resemble certain native pancreas features makes them promising candidates for the beta-cell-based studies. Here, as the first step, cellular heterogeneity and cell-cell contact in the pancreas are described to provide the basis for the cell types and the interactions in in vitro endocrine mimics of the pancreas. Next step involves an overview of the current field of the pseudoislets with the assembly techniques and their applications in pancreas biology and related fields.

## Cellular environment and communications of the pancreas

### Endocrine cells

**Fig. 1:**
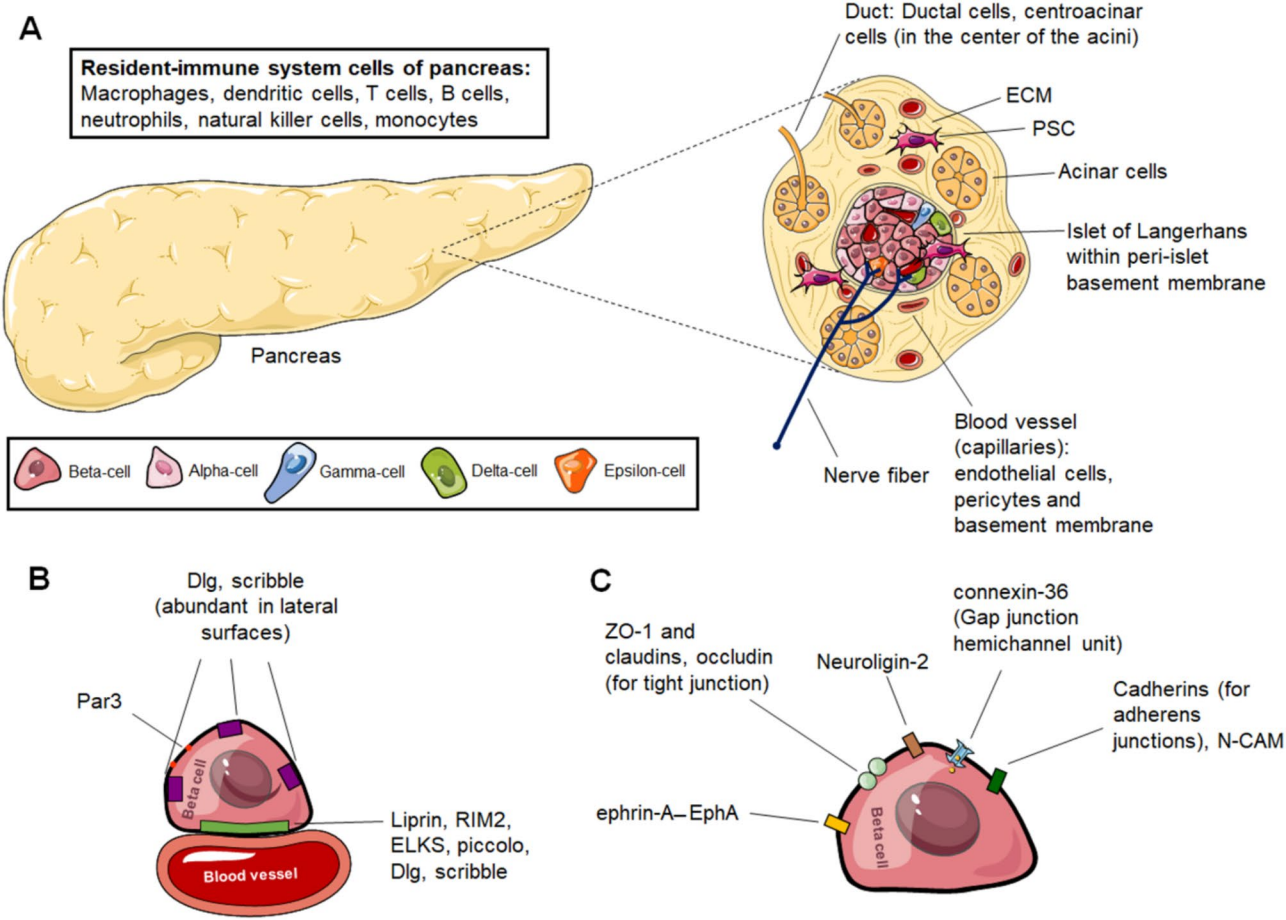
Organization in pancreas. **(A)** Pancreas, its compartments and microenvironment. Pancreas has two compartments: Endocrine and exocrine pancreas. Endocrine pancreas contains islets wrapped by peri-islet basement membrane. In islets, 5 different endocrine cells are present: Alpha-cells, beta-cells, delta-cells, epsilon-cells and gamma-cells. Exocrine pancreas is formed by acinar cells and ducts that contain ductal cells and centroacinar cells. In addition, resident-immune system cells and PSCs, as well as nerve fibers are found in the pancreas tissue. The pancreas also accommodates blood vessels. In figure as example, capillaries are shown. In general, capillaries are made of endothelial cells facing the blood and they are covered by pericytes and basement membrane. The acellular part of the pancreas is ECM that is rich in proteins, glycosaminoglycans and proteoglycans. **(B)** Polarity determinants for beta-cells. Beta-cells express Dlg and scribble in different regions, but their expression is enriched in lateral surfaces. Par3 is abundant in the apical domain away from vasculature. The capillary interface/vascular face is marked with the presynaptic scaffold proteins of liprin, RIM2, ELKS and piccolo. **(C)** Proteins on beta-cells used to form junctions for cell-cell interactions. ZO-1, claudins and occludin containing tight junctions, connexin-36-based gap junctions provide intercellular communication between beta-cells. Adherens junctions established between beta-cell and beta-cell and, between beta-cell and alpha-cell contain several cadherin family members and N-CAM. Apart from these junctions, EphA receptor tyrosine kinases and their ephrin-A ligands are located on beta-cells and alpha-cells for cellular communication and insulin secretion. Transcellular neuroligin-2 is an important protein of beta-cells to control insulin secretion. Dlg: Discs large protein, ECM: Extracellular matrix, N-CAM: Neural cell adhesion molecule, Par3: Partitioning defective 3 homologue, PSC: Pancreatic stellate cell, RIM2: Rab3-interacting protein. In labeling of endocrine cells for (A), the shapes and sizes of the cell representations are not relevant to the actual morphology and size of the cells. Figure 1 contains images from Servier Medical Art, which were modified in color and size where necessary. Servier Medical Art (smart.servier.com) is licensed under a Creative Commons Attribution 4.0 International License (CC BY 4.0) (https://creativecommons.org/licenses/by/4.0/)

An analysis of the pancreatic tissue architecture yields two compartments with distinct metabolic functions: Endocrine and exocrine parts. Endocrine part is crucial for the regulation of blood glucose and related metabolic activities [36]. In this section of the pancreas, islets of Langerhans (pancreatic islets) are the main cell clusters which are associated with hormone secretion. Cells in these clusters are mainly divided into five types (with core secretions): Alpha (α)-cells (glucagon), beta (β)-cells (insulin, C-peptide and amylin), delta (δ)-cells (somatostatin), epsilon (ε)-cells (ghrelin) and gamma (γ)-cells (pancreatic polypeptide) (Fig. 1A) [1, 37, 38].

Among these secretions, insulin has the central role in the glucose homeostasis and is reported to participate in the diverse mechanisms ranging from the bone growth to the endothelial cell function [39]. Besides to the role of insulin-copackaged amylin in the suppression of the glucagon secretion and reduced food intake, potential consequences of it in the adiposity, neurogenesis, nociception and maternal behaviors are suggested [40]. Although C-peptide is the one of the processing products of the preproinsulin, it has capacity to reduce certain adverse effects of the diabetes on the cardiovascular system, nervous system and kidneys [41]. Furthermore, it is possible to conclude that C-peptide is a promising target to reverse some severe side effect of the T1D, as the improvement of the beta-cell responsiveness to the hyperglycemia and the alpha-cell responsiveness to the hypoglycemia were recorded in T1D patients with the large residual C-peptide levels [42]. The other major hormone of the pancreas, glucagon, cooperates with insulin for the management of circulating blood glucose level, but its effect is opposite to insulin. It has actions such as the increase in the glomerular filtration, reduced food intake and rise in lipid oxidation in the muscles [43]. Somatostatin is another essential factor in the proper pancreas function that manipulates various processes in the central and peripheral tissues [44], as Li et al. demonstrated that the ablation of the somatostatin producing cells ends with the severe hypoglycemia and the neonatal death of the mice [45].

Overall, glycemic control is achieved by central and peripheral mechanisms. Postprandial blood glucose level activates glucose stimulated insulin secretion (GSIS) that inhibits hepatic glucose production through insulin receptor activation on hepatocytes and indirect inhibition of glucagon secretion from alpha-cells. GSIS also activates glucose uptake by tissues including muscle and adipose tissue. Activated by the nutrients, gastrointestinal intestinal tract endocrine cells release incretins that increase insulin release. In addition, the central nervous system contributes to the blood glucose management by hypothalamic-pituitary-adrenal axis and autonomic nervous system. In this mechanism, cortisol stimulates insulin secretion and sympathetic nervous system inhibits GSIS, stimulates glucagon release and hepatic glucose release. In contrast, parasympathetic nervous system can increase insulin and glucagon secretions, and activation of this system in postprandial conditions advances glucose uptake and storage [46–49]. Another hormone of islets, somatostatin, exhibits increased secretion when glucose level is elevated in the environment [50, 51].

An examination of the epsilon-cell derived ghrelin activity reveals distinct actions in the physiological processes, most notably on the glucose and energy metabolism [52]. In the body, the primary source of ghrelin is stomach, and it acts as “hunger hormone” by regulating appetite and controls various functions including sleep-wake cycle, lipogenesis and cardiac activities [53–55]. As a secretion of the islets, pancreatic polypeptide serves as a peptide hormone with the purposes such as the inhibition of the gastric emptying and sensitizing the liver to the insulin [56]. In addition to their systemic tasks, these islet hormones are destined for paracrine signaling. Somatostatin inhibits the insulin and glucagon release, and insulin negatively regulates glucagon secretion. Yet, glucagon is a known inducer for the insulin secretion and the insulin activates the somatostatin release [57, 58]. There is also evidence for observing no effect of insulin on somatostatin secretion. This discrepancy can be due to experimental models and species-dependent islet-cell organization [59, 60]. Expression of the ghrelin appears to be inhibited by the pancreatic polypeptide [56], but one of the key features of this hormone is the inhibition of the insulin and somatostatin [52].

Within the islets, localization and number of each cell type vary, as for instance, it has been previously reported that in the mouse islets, alpha-cells reside in the periphery whereas beta-cells occupy the central region. In contrast, human islets have alpha-cells localized throughout the islets, even making contacts with the intra-islet blood vessels [61]. Bosco et al. revealed that this cellular distribution is unique to the large human islets and, the smaller islets had similar core-mantle distribution as in the rodent islets, yet in cultured human islets, central localization of the beta-cells increases [62]. The dominant cell type for the human and the rodent islets is the beta-cell that accounts for at least 50% of the total population. Other abundant cell type is the alpha-cell with 38% and 18% of the endocrine cells in the human and mouse islets [61]. Within the population of the endocrine cells, transcriptional and functional heterogeneity exist which are reflected in the hormone secretion, protein profile, cell-cell communication, and response to the stimulators [63–69]. Soluble factor and ion-based cellular communication is universal for the islets, but also islet morphology is a key to achieve the proper insulin response to the glucose stimulation, as the islets dispersed into the single cells show an impairment of the insulin secretion due to destroyed direct cell-cell interactions [70].

Despite, in the T1D, beta-cells are destroyed by the immune attack, thus, at the time of diagnosis, up to 70–95% of the beta-cell reduction is accounted for the clinical symptoms. This beta-cell mass distribution is heterogenous among the patients and may not correspond to the severity of the disease, but significant loss occurs in T1D. In contrast, meta-analysis results indicate that the loss as low as 40% is sufficient to manifest the symptoms [71–73]. After beta-cell loss, changes in the alpha-cell behavior have been reported where controversial evidence is present in decrease/increase of alpha-cell number and glucagon levels [74, 75]. Basically, this injury of the beta-cells enriches alpha-cells and causes functional impairment. These alpha-cells lose the expression of alpha-cell markers of MAFB and ARX, as well as they start to express the beta-cell marker NKX6.1. Furthermore, the events leading to the alpha-cell related complications depend on the changes in the expression of ion channels, cAMP-mediated pathways, and vesicle trafficking proteins. Thus, electrical activity as well as glucagon release weaken [76, 77]. To counteract the effects of the lowered beta-cell abundance, a recent study has shown that immunostained alpha-cells are positive for the insulin and the glucagon with PDX transcription factor expression in the diabetic mice [78]. In contrast, another possibility includes that MAFB and ARX marker transcriptions are preserved, and the genes for the glucagon biosynthesis and processing are upregulated in the T1D patients [79].

Although low in number, delta-cells are central to regulate the endocrine dynamics of the pancreas. Equipped with filopodia, these cells can reach out not only the beta-cells in close-proximity, but also distant beta-cells in the islets and exocytose the somatostatin from the soma and the filopodia. In an attempt to contact with the beta-cells, filopodia length increases in the prediabetic delta-cells [80]. In the childhood and adult onset diabetes, the number of alpha- and delta-cells are reported to be constant while due to a major loss of the beta-cells in the head and tail regions, islet size was measured to be smaller [81]. The islet size and size distribution can also remain similar to the healthy pancreas, but significant reduction in the islet density of recent onset T1D and long-standing T1D occur [82]. Previously, a significant improvement in the gamma-cell % in the islets was noted in the T1D patients [83]. Consequently, alpha-cell and delta-cell content may vary in the T1D that eventually alters other endocrine activities of the pancreas.

### Cell-cell contact based signaling in the islets

Beta-cells are polarized to develop appropriate responses in the islets, as they express basolateral polarity determinants of discs large protein (Dlg) and scribble in different regions, but these proteins are enriched in lateral regions and, partitioning defective 3 homologue (Par3) is localized in the apical domain away from the vasculature. The capillary interface/vascular face is marked with the presynaptic scaffold proteins of liprin, Rab3-interacting protein (RIM2), ELKS and piccolo (Fig. 1B) [84–88]. These proteins guide beta-cells to position themselves towards the vasculature and in fact, the polarization of beta-cells is important for the secretory behavior. For instance, glucose transporter (GLUT)-2 is laterally positioned and zona occludens (ZO)-1 containing tight junctions are in the apical domain showing that the polarization can be a directing clue for the secretion [84]. Experiments on the mice and in vitro tests show that ELKS participates in the polarized insulin secretion via L-type voltage-dependent calcium channel (VDCC) opening and first-phase insulin secretion through direct binding to the VDCC-β subunit. These events occur at the vascular face of the beta-cells where ELKS is contained [85].

Gap junctions (Fig. 1C) are exceptional intercellular channels which provide coupling of two cells metabolically and electrically. Islets express gap junction subunits called connexins and among all, connexin-36 has outstanding effects on the beta-cells [89, 90]. These gap junctions connect beta-cells, especially connexin-36 based junctions are functional in the electrical coupling and promoting the protection of the beta-cells against cytokines and streptozotocin (STZ). Recently, Farnsworth et al. demonstrated that the gap junction coupling decreases at the T1D onset and this change along with the modifications of Ca^2+^ amplifies the susceptibility to apoptosis. Yet, connexin-36 gap junction coupling in NOD mouse can be protective against the early onset of the T1D without progress in the long-term survival, islet function and beta-cell death [91]. In the cell-cell contact, gap junction coupling of the beta- and delta-cells is reported to have behavioral consequences, as the beta-cell signaling through these junctions controls the delta-cell [Ca^2+^]i fluctuations and somatostatin secretion [92]. Beta-cells also contain channels called pannexin-1 which are similar to the gap junction hemichannels, but they release ATP [93]. This extracellular ATP binds to receptors such as P2X purinoceptor 7 (P2X7R) mobilizing Ca^2+^ towards the intracellular pathway to secrete the insulin [94].

Adherens junctions established with E- and N-cadherins contribute to the beta-cell functionality with several potential mechanisms (Fig. 1C) [95]. In polarized beta-cells, catenins and cadherins control the insulin granule pool, as the cadherin or p120 catenin knockdown shows a trend towards increased basal insulin levels. This is achieved by the cadherin mediated downstream signaling of the catenins where a possible role of p120 catenin in the actin network remodeling along with the α- and β-catenins involved, and that the glucose increases the expression of cadherin and p120 catenins. Thus, E- and N-cadherins promote GSIS from the single beta-cells [96, 97]. Human islets express E- and N-cadherin, which are mostly present on the beta-cells with some positivity on the alpha-cells for N-cadherin. This cadherin expression pattern in the cellular interactions can sustain the islet viability. In fact, Parnaud et al. proved that the adhesion of the single beta-cells to the cadherin substrate is directly involved in the protection against cytokine induced and general apoptosis [98]. Desmoglein-2 is another member of the cadherin family that shows expression on various cells within the pancreas, including the cells within the islet (highest in the beta-cells), the vasculature and the exocrine tissue. Similar to E-cadherin, it can participate in the protection against STZ-induced cell death in vivo and cytokine induced apoptosis in vitro [99]. As a member of Ig superfamily, neural cell adhesion molecule (N-CAM) is expressed in the islets for the control of cell segregation, insulin and glucagon exocytosis management through the actin reorganization coupled with the cadherin activity [100–102].

Cadherins also contribute to the native islet organization in the pancreas organogenesis. In pancreatic development, E-cadherins of beta-cells are essential to cluster these cells into islets. For instance, expression of truncated E-cadherin disrupts assembly of beta-cell into aggregates and alpha-cells organize into islet-like clusters without beta-cells [103, 104]. Besides, this direct cell-cell contact provided by E-cadherin lowers beta-cell proliferation [105, 106]. In islets, the endocrine cell segregation is affected by expression of cadherins and N-CAM. It is suggested that these cell surface molecules create alterations in surface tension leading to localization of each cell type into distinct regions. Loss of one cadherin can be compensated by others, but loss of N-CAM for instance, results in alpha-cell and beta-cell mixing and cadherin redistribution [107]. Further, at ∼E10.5, cap cells contain less E-cadherin and this lowered amount is associated with the start of pancreatic branching and tip formation [108].

Various EphA receptor tyrosine kinases and their ephrin-A ligands are expressed by the pancreatic islets and the beta-cells (Fig. 1C), and EphA forward signaling inhibits the insulin secretion while ephrin-A reverse signaling stimulates the insulin secretion [109]. In T1D, some of the juxtacrine signaling such as ephrin-A – EphA4/7 between the beta-cells and the alpha-cells are most probably lost relieving the F-actin based inhibitory effect of this interaction on the alpha-cells, further contributing to the alpha-cell dysfunction in the T1D [110]. Finally, claudins, occludin, and ZO-1 as the tight junction units expressed in the islets (111, 112]are important. For example, claudin-4 is associated with the maturation and dedifferentiation of the beta-cells [111].

Some proteins in the inhibitory synapses of the central nervous system have been discovered to be expressed in the islets with the capacity to manipulate insulin secretion. Transcellular neuroligin-2 is reported to be an insulin release stimulator as indicated in a co-culture system experiment where beta cells/islet cells were seeded on HEK293 cells transfected to express full-length neuroligin -2. Underlying mechanism for this effect includes reduction of the insulin granule docking as shown in the pancreatic islets of neuroligin-2 knock-out mice (33% decrease). Its common partner, neurexin, is shown to be dispensable from this process raising the question of the presence of other unassessed interaction partners on the beta-cells [113].

### Islet capillaries and cell-cell communication

Pancreatic islets are rich in blood vessels to deliver the hormones to target cells as well as to sustain themselves [114, 115]. The intra-islet capillaries are formed by the blood contacting endothelial cells with a cover of pericytes and a vascular basement membrane. Further, the whole islet is wrapped with the peri-islet basement membrane isolating the islet from the surrounding tissue [116–118]. This morphological assembly as well as its organization within the islets are useful tools to deliver insulin to the endothelial cells.

One of the primary controllers of insulin secretion from beta-cells is the high glucose concentration in the bloodstream. Following glucose transport into the pancreatic beta-cells through glucose transporters (GLUT-1 and 3 human beta-cells and GLUT-2 for rodent beta-cells), glucose is phosphorylated by glucokinase and is delivered to glycolysis pathway [119]. Energy level as ATP amount generated by this pathway as well as TCA cycle with upcoming oxidative phosphorylation increase ATP/ADP ratio intracellularly. This stimulus activates ATP-sensitive potassium (K_ATP_) channels to close leading to membrane depolarization. In turn, this electrical alteration in the membrane charge opens voltage-gated Ca^2+^ channels of beta-cells resulting in high Ca^2+^ influx into the cell. Next stage involves exocytosis of insulin granules from readily releasable pool with profound insulin increase in the blood. Another internal storage pool of beta-cells is also induced to sustain insulin level postprandially resulting in biphasic release. Anaplerotic pathways for TCA cycle intermediates, amino acids (e.g. lysine, glutamine, alanine), pentose monophosphate shunt and fatty acid metabolism further fuel the granule release [37, 120–124]. The released insulin travels through fenestration of the endothelium and by caveolae-mediated transendothelial transport and reaches blood circulation [125]. To facilitate the transfer process, density of the fenestrae in the islet capillaries are much higher than the exocrine tissue to deliver the hormones and other molecules to the blood circulation [126, 127].

At physiological concentration of insulin, endothelial cells uptake this hormone from vessel lumen through receptor mediated endocytosis with the insulin receptors [128–130]. Other possible routes also exist for insulin transport by endothelial cells such as receptor-mediated endocytosis (insulin receptor and insulin-like growth factor-1 receptor), fluid-phase endocytosis [129] and transcytosis [131, 132]. For this reason, the beta-cells mostly contact with the capillaries [133].

In the blood vessels, endothelial cells are connected to each other through transmembrane proteins called junctions. These endothelial cell junctions provide contact points between two cells and regulate processes including vessel permeability and stability, endothelial cell continuity, mechanosensation, leukocyte diapedesis, and inhibitory signaling for the proliferation [134–139]. The capillary junctions are mainly classified into two groups: Adherens and tight junctions. At adherens junctions, vascular endothelial cadherins (VE-cadherins) originating from the distinct membranes link two endothelial cells. In contrast, at tight junctions, adhesion is controlled by the claudin family (claudins 1, 5 and 12), occludin, junctional adhesion molecules (JAMs) and endothelial cell selective adhesion molecule (ESAM). Nectin–afadin complex is reported to contribute to both junctions. Platelet endothelial cell adhesion molecule-1 (PECAM-1) is another junction unit that connects the endothelial cells [139, 140].

Additionally, endothelial cells and beta-cells are coupled physically and through soluble factors. Connexin-43 of the endothelial cells and connexin-36 of the beta-cells are suggested to form the connexon connecting these cells [102]. Endothelin-1, hepatocyte growth factor, thrombospondin-1 and connective tissue growth factor secreted by the endothelial cells stimulate insulin release, serve in the development of the pancreas and support the beta-cell viability. Similarly, factors such as insulin, vascular endothelial growth factor A (VEGF-A) and angiopoietin (ANG)-1 of the beta-cells positively regulate the endothelial cell survival and physiological activities [141–144]. As an effective way to communicate with the neighboring cells, islets also produce extracellular vesicles. The surfaceome of these vesicles include alpha6- and beta1-integrin, CD44, ICAM-1 (intercellular adhesion molecule-1), CD31 and glucagon-like peptide-1 receptor (GLP1R) and the constituent molecules of these vesicles are such as insulin, C-peptide, CD63, programmed death-ligand 1 (PD-L1), Argonaute-2 and proteins of cell proliferation. Besides, distinctive cellular metabolic alterations occur in the endothelial cells, as these vesicles are equipped with various mRNAs for the endothelial cell activation and angiogenesis, insulin production and signal transduction, glycogen/lipid/protein synthesis and peroxisome proliferator-activated receptor pathway. Moreover, they are enriched with miRNAs such as miR-375, miR-126 and miR-296. Thus, upon transfer to the endothelial cells, pro-angiogenic (ANG-1, VEGF-alpha, vascular endothelial growth factor receptor (VEGFR) -1, -2) and anti-apoptotic factors (Bcl-2) are upregulated, and anti-angiogenic (thrombospondin1) and pro-apoptotic molecules (BAD) are downregulated pointing out the anti-apoptotic and angiogenic effects of the islets on the endothelial cells [143, 145–147].

Pericytes as mural vascular cells in islet capillaries cover 40% of the vasculature and, under sympathetic adrenergic control, they contract the blood vessels altering capillary diameter and flow. Conversely, high glucose in the circulation inhibits pericytes, thus dilation and high blood flow occur. These cells also aid beta-cell function and density [148–150]. They control beta-cell activity as well as the establishment of both islet basement membrane and interstitial matrix, as they secrete collagen IV, laminins (especially α2 and α4 laminins), proteoglycans, fibronectin, nidogen, and hyaluronan [118]. During T1D, as these cells become dysfunctional and they presume a myofibroblastic phenotype, coverage of the vessels by these cells decreases. These events trigger the impairment of the balance between vasoconstriction and vasodilation in the islet capillaries which affects the islet perfusion, possibly creating a hypoxic environment that activates the expression of the certain vascular modeling genes such as the vasodilator peptide adrenomedullin, migration and metabolism genes in the islets [150]. Additionally, islet vascular density has been significantly built-up in the T1D patients, as upregulation of the genes associated with the vasculature and angiogenesis whereas downregulation of the epithelial-to-mesenchymal transition genes have been reported. Interestingly, these islet blood vessels are shorter and smaller in diameter compared to the healthy pancreas capillaries [151, 152].

In general, as pericytes wrap around the capillaries, endothelial cells and pericytes present cell-cell contact mediated interactions and soluble factor signaling to regulate the healthy vessel activity. These cells interact with the extracellular matrix (ECM) and, through the regions called adhesion plaques and peg-and-socket interactions to manipulate the mechanical and chemical sensing in different regions in the body. At the cell-cell contact intersection, adhesion plaques and peg-and-socket regions closely connect two cells. Adhesion plaques are rich in fibronectin and anchor the pericytes to the bottom epithelium whereas connexin-43 based gap junctions and N-cadherin mediated adherens junctions are in the peg-and-socket regions. In these regions, various soluble factors and their corresponding receptors are also localized. For instance, VEGF-A and VEGFR-1, platelet-derived growth factor (PDGF)-BB and platelet-derived growth factor receptor-β (PDGFRβ), ANG-1 or ANG-2 and Tie2, Notch3 and Jagged1, Eph-B2 and Ephrin-B2 and, transforming growth factor (TGF)-β and transforming growth factor-β receptor (TGF-βR) communications develop for the diverse applications from the quiescence to the perfusion [153–156].

### Exocrine pancreas and cell-cell contacts

Exocrine pancreas is responsible for the digestion process, since it secretes enzymes such as amylase, pancreatic lipase and trypsin into the pancreatic ducts [1, 38]. In this part of the pancreas, acinar cells produce and secrete digestive enzymes with a NaCl supplied fluid. Ductal cells are arranged as an epithelial lining to form intercalating, intralobular and interlobular ducts directing the enzymes to the duodenum. Ductal cells are also the sources of the protective layer of the mucus. An extension of the duct lined by the centroacinar cells reaches the center of the acini at the end of the intercalating duct. The ducts are responsible for the secretion of bicarbonate along proximal to distal ducts to prevent the enzyme aggregation while Cl^−^ concentration decreases [157–160]. Unlike the healthy pancreas, T1D exocrine pancreas is damaged due to the abnormal metabolic transformation. Pancreatic amylase, lipase, retinol binding protein and prealbumin decrease in the long-standing T1D patients reflecting the outcome of this process [161]. The level of exocrine enzymatic activity such as lipase and trypsinogen are proposed to be serological markers in the staging of T1D and the size changes in the pancreas [162]. Significantly lower number of the acinar cells occupy the T1D patient pancreas [163] whereas no acinar atrophy is observed in some other patients, yet a possible link to the pancreatic cancer is established [164]. Further, these cells can be reprogrammed into beta-cells. On the basis of the recent observations, the findings on the ductal cell genetic reprogramming through Neurog3, MAFA, PDX1 and PAX6 for sourcing beta-cells showed encouraging therapeutic implications for the insulin-secreting cell transplants [165].

Primary gap junction hemichannels on the acinar cells are based on the connexin-32 and  -26 [166, 167]. The evidence regarding the necessity of these junctions for the physiological function is obtained from, for instance, homozygous connexon-32 deficient mice which displayed increased enzymatic output (e.g. amylase) compared to the controls. This model paved the possibility of the inhibitory effect of the gap junctions on the number of mobilized acinar cells for the enzyme secretion, as upon release of this effect through the loss of connexon-32 channels, the acinar cell number and the amylase release are improved [168]. Similar to the endocrine pancreas, E-cadherin is expressed in the exocrine part and the loss of it severely disturbs the exocrine pancreas architecture and the downstream pathways such as Wnt signaling and Hippo/YAP signal transduction pathway, and the mutant acinar cells undergo the acinar-to-ductal metaplasia [169]. The presence of the tight junctions of the ductal cells regulates the ductal polarity and the paracellular transport which are crucial for the functional state of the ductal cells. Molecular composition of the ductal and acinar tight junctions includes the claudin family members such as claudin -1, -4, -7, occludin, JAM-A, ZO-1, ZO-2, and tricellulin [170–172].

### Immune system cells and others

Remaining space available for the cells in the pancreas is occupied by other cell types such as pancreatic stellate cells (PSCs). PSCs are quiescent, vitamin A-rich lipid storing cells in the pancreas. They are precursors for the cancer-associated fibroblasts (CAFs) and in their activated state, they presume myofibroblast-like phenotype. Along with the CAFs, PSCs secrete ECM proteins (e.g. fibronectin, collagen I, hyaluronan) and other soluble factors (e.g. TGF-β, IL-1β, IL-6, leukaemia inhibitory factor (LIF), PDGF, VEGF), as well as release extracellular vesicles with the nucleic acid and other molecules (e.g. miR-5703, miR616-3p, miR-4456, lactate, glutamine, threonine, phenylalanine, stearate, palmitate) in the diverse stages of the cancer [173–176]. For instance, fibronectin deposited by the PSCs promotes gemcitabine resistance through ERK1/2 phosphorylation [177]. During T2D, insulin fuels activated PSC growth and ECM deposition resulting in the massive fibrosis [178]. In diabetic conditions, hyperglycemia, hyperinsulinemia and renin–angiotensin system activate PSC proliferation and fibrotic activity. Interference of these pathways is suggested to improve the treatment outcomes in the clinics [179, 180]. Furthermore, activated PSCs can cause beta-cell exhaustion [181]. However, PSCs are reported to act as a determinant to sustain the islet cell phenotype and survival in vitro as well as promote islet graft activity in vivo. In a recent study, it is proposed that activated PSCs can return to the quiescent state after transplantation and, the nonfibrogenic effect of these cells should be considered. Direct co-culture experiments where islets were seeded on top of PSCs suggest that interactions with PSCs improved islet function and viability, slowed down morphology loss and sustained islet markers of insulin, PDX and glucagon. In the same study, PSCs transplantation with syngeneic islets showed small but significant decrease in blood glucose level, and oral glucose tolerance tests showed improved glycemia and insulin secretion function of islets showing their potential to reach normoglycemia [182]. Activated PSCs were reported to support exocrine cell differentiation in human pancreas [183]. In autoantibody^+^ patients, fibronectin, periostin and collagen subunit encoding genes are expressed largely in the stellate cells, yet T1D patient stellate cells are negatively affected, and their phenotype shifts to the immunomodulatory one [150]. Contribution of these cells to the development of pathologies such as pancreatic cancer and pancreatitis is also well-documented with soluble factors, extracellular vesicles, ECM remodeling and synthesis, and resistance to chemotherapeutic drugs and inhibition of anti-tumor response of immune system can be built-up by PSCs [184–195]. Moreover, among PSCs, functional heterogeneity promotes pancreatic cancer development as both subgroups, myofibroblastic and inflammatory PSCs, complement each other to progress pancreatic cancer [193].

In a healthy body, immune system protection against the pathogen invasion is indispensable from the survival and this network also assists homeostasis. To accomplish these, various tissues maintain resident immune system cells and upon requirement, migrating members strengthen the defense, thus physiological function. One of the resident constituents of the immune division in the pancreas is macrophages which are observed in both endocrine and exocrine sections. They make up 98% of the islet-resident CD45^+^ cells in the mice [196, 197]. Pancreatic macrophages are derived from the yolk sac and the bone marrow and play dual roles in physiological situations and transition into the pathological state. Polarization of the macrophages is the fundamental concept to determine their action in the beneficial-detrimental spectrum. Polarized macrophages can presume M1 or M2 phenotype depending on the factors they are exposed to. In a healthy pancreas, both sets are present and in fact, these macrophages do not strictly follow the definition of M1 and M2 macrophages. Macrophages in the pancreas are main suppliers of IL-1β, IL-6, IL-12, TNF (tumor necrosis factor)-α, IFN (interferon)-γ and nitric oxide (NO) which are largely responsible for the beta-cell dysfunction/death and the insulin resistance. Nevertheless, other macrophages generally express large amounts of IL-10, TGF-β1, epidermal growth factor (EGF), hepatocyte growth factor (HGF), PDGF, insulin-like growth factor (IGF)-1, matrix metalloproteinases (MMPs) and Arg1 to protect and regenerate the beta-cells, for the pancreas organogenesis and the insulin sensitivity [197–202]. In return, islets modulate macrophage monitoring by shaping the pseudopodia formation [203].

In the diseased state, macrophage derived factors such as RANTES and TNF-α genetically reprogram acinar cells contributing to the progression of the acinar-to-ductal metaplasia [204]. Another aspect of paracrine signaling involving macrophages activates PSCs during fibrotic growth [205]. Besides to their role in the T cell activation and autoantigen presentation [198], macrophages secrete CXCR2 (CXC chemokine receptor 2) ligands as well as induce beta-cells by IL-1β to secrete CXCL (C-X-C motif chemokine ligand)1 and CXCL2 to recruit the neutrophils to the pancreas in T1D [206]. Although macrophages have detrimental effects on the onset and progression of diabetes, they exercise beneficial activities for the pancreas. As described by Xiao et al., M2 macrophages interact with the beta-cells through TGF-β1 and EGF signaling. Signaling by these soluble factors triggers SMAD7-CyclinD axis and exclusion of p27 from the nucleus which switches on the beta-cell proliferation. However, another branch of TGF-β1 pathway that activates SMAD2 is inhibitory on the beta-cells. The countering route mediated by EGF activation inhibits SMAD2 activity [207]. Further, macrophages are advantageous actions for the beta-cells such as islet remodeling, vascularization and insulin secretion [208]. All these data indicate that support and attack are both modulated by macrophage-derived factors and their signaling pathways in T1D.

Dendritic cells are antigen presenting cell arm of the hematopoietic cells between innate and adaptive immune system in the multicellular organisms. Tissue-resident subgroup of the dendritic cells in nonlymphoid tissues often migrate to the lymph node to induce T-cell activity [209]. In the pancreas, they are localized adjacent to the intra-islet blood vessels, mostly between the vessel wall and the beta-cells. The dendrites of these cells are highly active moving within the beta-cells for screening. Dendritic cells engage with the beta-cell derived antigens for MHC-dependent presentation to the T cells in the healthy pancreas suggesting role of this dendritic cell activity requiring further investigation in the development of the autoimmune diseases [210]. In the acute pancreatitis of mouse, dendritic cells secrete proinflammatory cytokines, activate adaptive immune system as well as participate debris and by-product removal to support the pancreatic viability [211].

Among lymphoid lineage, CD3^+^ T cells are the most abundant being 79% of this population and, CD8^+^ cells constitute 78% of T cells most of which are memory cells in the islets [212]. As main players in the defense against pathogens, establishment of memory functions, immunoregulation and screening for cancer cells, T cells regulate homeostasis in the body [213] and during ageing, T cells accumulate in the non-diabetic mouse pancreas [214]. In the pancreas, PD-L1 (Programmed death-ligand 1) – Programmed cell death protein-1 (PD-1) binding between macrophages and CD8^+^ memory T cells regulate immune homeostasis [215] as well as these cells represent 43% of CD8^+^ T cells in the insulitis lesions in the T1D [216]. 8–20% of CD4^+^ T cells in the pancreas are Tregs which are the suppressive cells to allow immune-tolerance and, have distinct subsets secreting IL-10 and TGF-β, preventing inflammation and suppressing the Th1 inflammation. Tregs in the pancreatic lymph nodes secrete the same factors and perform the same tasks, but also secretion of IFN-γ is noteworthy [217]. Occasionally, natural killer cells and B cells are found in the pancreas [212]. These group of cells (dendritic cells, monocytes, B cells, T cells and innate-lymphoid cells) also exist in the exocrine pancreas [197].

Finally, the involvement of the nervous system to the pancreas activity should not be underestimated. The enrichment of the parasympathetic afferent fibers in the pancreas participates in the mechanical and chemical sensing in homeostasis, pain and inflammation. Besides, sympathetic afferent nerves are used for mechanical and chemical sensing by transmitting the signals from vasculature, acini, islets and intrapancreatic ganglia. The efferent network is directly involved in the pancreas activity. For instance, factors such as vasoactive intestinal polypeptide and gastrin-releasing peptide secreted by the nerve fibers manipulate the insulin secretion [218, 219]. Moreover, sympathetic neurons cooperate for the proper islet assembly, GSIS and the beta-cell migration during the development [220].

### Scaffold-free strategies for the design of an artificial islet

Considering the etiology of T1D, cell-based therapy and experimental models aim to simulate the native environment as well as seek strategies to advance the insulin secretion, lower cell/transplant number, find alternative insulin-secreting cell sources and provide companion cells to stimulate the viability and the vascularization. These can be achieved by the co-transplantation [221], cellular coating [222, 223], differentiation/alternative beta-cell sources [224, 225], development of ECM mimics [226–228] and immunoisolation barriers [229–232]. One of the other largely studied alternative strategies is multicellular assemblies which can achieve the targeted aims with only cells and when necessary, in combination with genetic engineering. This innovative approach unlocks various applications from disease modeling to cell/drug based T1D therapy (Table 1) [233, 234].

**Table 1:** Multicellular beta-cell assemblies and their applications. CCL22: C-C motif chemokine ligand 22. CHPOA: Acrylate modified, cholesterol bearing pullulan. ECM: Extracellular matrix. GLP-1: Glucagon-like peptide-1. GPCR: G-protein coupled receptor. hiPSC: human induced pluripotent stem cell. IL: interleukin. MBP-FGF2: Maltose-binding protein-basic fibroblast growth factor 2. MSC: Mesenchymal stem cell. PEG: Poly(ethylene glycol). PEI: Poly(ethyleneimine). PDMS: Polydimethylsiloxane. PNIPAAm: Poly(N-isopropylacrylamide). PMMA: Poly(methyl methacrylate). STZ: Streptozotocin. T1D: Type 1 diabetes.

Nomenclature	Technique	Cell type/source	Main studied aspect(s)	Ref.
Spheroid	• 100 mm^2^ non-adherent plastic dish • ECM gel (laminin, collagen type IV, heparin sulfate proteoglycan, entactin)	MIN6	Spheroids as models to control unlimited growth after transplantation	[235]
Spheroid	PDMS-based concave molds coated with bovine serum albumin	Dissociated rat islet and hepatocyte	Relationship between islets and hepatocytes for cell-based liver or T1D therapy	[236]
Spheroid	Hanging drop	Dissociated rat islet	Assembly of nonviral PEI–mediated IL-10 cytokine gene-delivered cells into spheroids	[237]
Spheroid	3D clinostat (microgravity)	MIN6	Optimization of spheroid properties for transplantation potential	[238]
Pseudoislet Spheroid	96-well non-adhesive prime surface plate	α-TC1 clone 6, NIT-1 and TGP52	Effects of seeding density and cell type ratio on cell distribution, hormone mRNA level and functionality	[239]
Spheroid	6-well plate with PNIPAAm-coated PDMS-based microwells	NIT-1	Therapeutic potential in STZ-induced diabetic mouse	[240]
Spheroid	PDMS-based microwell coated with PNIPAAm	MIN6 and MAEC MIN6 and NIH3T3	Encouragement of insulin secretion induction with companion cells	[241]
Spheroid	100-mm tissue culture dish	INS-1E expressing insulin-GLase	Example case to analyze protein secretion via video-rate bioluminescence imaging	[242]
Spheroid	Layer-by-layer coating with fibronectin and gelatin followed by low cell attachment 96-well U-bottom plate	MIN6	Cell coating for structural stability and functionality of spheroids in T1D treatment	[243]
Spheroid	Membrane-bottomed PDMS concave microwell array	Dissociated rat islet	Development of a novel system to overcome oxygen supply limitation for functional and viable spheroid formation	[244]
Spheroid	Microfluidic chip containing hemispheric cell culture chambers and with methyl cellulose and collagen type I containing medium feed	Human pancreatic 1.1B4	Development of a platform with perfusion flow network that enables beta-cell spheroid assembly and measurement of beta-cell function under dynamic conditions	[245]
Spheroid	Ultra-low attachment 100 mm dish	MIN6-K20	Model along with monolayer culture in the study of incretin responsiveness state of beta-cells	[246]
Spheroid	Geometry-controlled hanging drop	βTC6	Effects of droplet spreading on the spheroid formation and function	[247]
Spheroid	Microfluidic chip containing PDMS-based concave microwells	Dissociated rat islet	Comparison of static culture with dynamic culture, drug testing (tolbutamide, GLP-1, rapamycin)	[115]
Spheroid	PDMS microwell coated with PNIPAAm or collagen I	MIN6 and NIH3T3	Alteration of cell localization by control of migration	[248]
Spheroid	PDMS-chip and PMMA-chip	MIN6, MIN6-m9	Study of the role of oxygen supply and antioxidant mediated minimization of reactive oxygen species during bioartificial pancreas preparation	[249]
Spheroid	Hanging drop	MIN6 and rat bone marrow MSC	Development of heterospheroid model for functional and survival boost combined with PEG hydrogel as immunoisolation barrier	[250]
Spheroid	Concave microwell coated with bovine serum albumin	Human liver cells transdifferentiated into insulin producing cell	Spheroid size control, improvement of actin–myosin-associated cytoskeleton changes mediated differentiation and function in vitro and STZ-induced diabetic mouse	[251]
Spheroid	• 35 mm non-adherent petri dish • Hanging drop • Agarose 3D microwell technique • Spherical plate 5D	Dissociated rat islet	Comparison of different spheroid assembly techniques for obtaining optimum islet mimics	[252]
Spheroid	Honeycomb polygons of PDMS	hiPSC	Cellular and functional characterization, effects of dimension, potential to establish pancreas-on-chips	[253]
Spheroid	PDMS honeycomb sheet coated with pluronic acid	hiPSC	Transcriptomics and metabolomics profile comparison of spheroids in culture of 3D static vs. microfluidic chip	[254]
Spheroid	Stirred-tank bioreactor	INS-1	Effects of technique and stirrer type in the assembly, viability, functionality and yield	[255]
Spheroid	Ultra-low binding round-bottom plate as part of differentiation process MBP-FGF2 immobilized surface as part of differentiation process	Human omentum-derived stem cell	In vitro differentiation in the spheroid formation platform, characterization, and analysis of therapeutic potential in STZ-induced diabetic mouse	[256]
Spheroid Organoid	Amikagel containing 96-well plate during aggregation following final differentiation	Human embryonic stem cell derived pancreatic progenitor cell and HUVEC	Development and validation of a hydrogel-based system for progenitor cell differentiation towards pancreatic lineage and organoid assembly	[257]
Spheroid Organoid	Agarose-patterned microwell	Dissociated islet and human amniotic epithelial cell	Enhancement of engraftment, viability, and graft function in STZ-induced diabetic mouse	[258]
Organoid	Microfluidic chip with microwell configurations as part of differentiation process	hiPSC lines	iPSCs as a source of insulin-producing beta-cells for diverse uses	[259]
Organoid	Agarose-coated 96-well plate	Dissociated mouse islet and mouse microvascular fragment	In vitro characterization and development to address challenge of insufficient vascularization after islet transplantation	[260]
Organoid (or pseudoislet)	Ultra-low attachment 96-well microplate	Dissociated mouse islet	Assessment of spheroid potential for genetic manipulation with adeno-associated virus serotype 8	[261]
SC-islet (organoid)	Ultra-low attachment cell culture plate on orbital shaker	Human H1	Model beta-cell system for the development of the reverse phase liquid chromatography-tandem mass spectrometry for insulin detection	[262]
Pseudoislet	Tissue culture flask coated with gelatin	MIN6	Potential of E-cadherin on the regulation of pseudoislet growth	[263]
Pseudoislet	90 mm bacterial (uncoated) petri dish	MIN6	Development of an immunoisolation barrier for cell-based therapy in T1D	[264]
Pseudoislet	Uncoated 90 mm petri dishes	MIN6 and mouse MSC	Testing layer-by-layer nanoencapsulation system on the spheroids as models for immunoisolation	[265]
Pseudoislet	6-well tissue culture plate	βTC3 and MS1	Effect of dimension and endothelial cells on function, ECM synthesis and viability	[266]
Pseudoislet	Ultra-low attachment, 6-well, flat-bottomed plate	Human 1.1B4	Evaluation of pseudoislets as alternatives to natural islets for transplantation to STZ-induced diabetic mouse	[267]
Pseudoislet	Agarose gel-based microwell	Dissociated rat islet	Characterization to investigate usefulness of pseudoislets for transplantation into STZ-induced diabetic mouse	[268]
Pseudoislet	2D clinostat (microgravity) followed by culture in 6 cm petri dish	EndoC-βH3	Description of response to hypoxia	[269]
Pseudoislet	Hanging drop	MIN6	Optimization of alternative beta-cell model and development of CHPOA nanogel coating for immunoprotection	[270]
Pseudoislet	96-well spheroid microwell plate	Dissociated human islet	Characterization including cellular heterogeneity and assessment of spheroid potential for genetic manipulation with lentivirus	[271]
Pseudoislet	Centrifugal forced aggregation in 24-well or 6-well AggreWell 400 plate	Dissociated human islet	Potential to be an alternative for native islets and superiority upon transplantation in STZ-induced diabetic mouse	[272]
Pseudoislet	96-well ultra-low adherent culture plate	Alpha-, beta- or delta-cells from dissociated human islet	Addressing beta-cell source limitation in the replacement therapy by reprogrammed alpha- and delta-cells	[273]
Pseudoislet	Hanging drop	Dissociated human islet	Study of effects of glucose, adrenaline and palmitate on the regulation of insulin and glucagon secretion	[274]
Pseudoislet	Hanging drop (GravityPLUS)	Dissociated mouse islet	Assessment of spheroid potential by adenovirus-delivered V1b receptor overexpression in vitro and STZ-induced diabetic mouse	[275]
Pseudoislet	6-well tissue culture plate	βTC3 and MS1 MIN6 and MS1	Effect of dimension, endothelial cells and secretagogues on function and gene expression	[276]
Pseudoislet	Ultra-low attachment microplate Hanging drop (GravityPLUS)	Dissociated human islet	Spheroid cell content, viability, functionality (dynamic perfusion and microfluidic system) analysis to develop representative model in GPCR signaling analysis	[277]
Pseudoislet	96-well plate with a non-adherent round bottom	EndoC-βH3	Monitoring and analysis of endocrine activity in the dynamic system of a microfluidic chip that allows self-trapping of pseudoislets	[278]
Pseudoislet	Suspension flask with stirring	INS832/3	Characterization of insulinoma pseudoislets	[279]
Pseudoislet	Agarose-coated microwell array	αTC1 clone 6, INS-1E and HUVEC	Analysis of cell distribution within spheroids	[280]
Pseudoislet	PDMS microfluidic system with microtrap chambers	α-TC1 clone 6 and INS-1E	Islet-on-a-chip microfluidic 3D model for mimicking in vivo for spheroid assembly and for drug test platform for palmitic acid hydroxy stearic acid	[281]
Pseudoislet	Low attachment microwell plate coated with pluronic acid	αTC1 clone 6 and INS-1E	Model in the discovery of the compounds effective on the reduction of oxidative stress with viability and functionality assays	[282]
Pseudoislet	96-well plates with V-bottom conical wells pre-treated with anti-adherence rinsing solution	Mouse and human FAC-sorted beta-cell and HUVEC	Analysis of impact of beta-cell CD63 expression in transplantation	[283]
Pseudoislet	AggreWell 400 6-well plate followed by centrifugation and shaking, transfer to an ultralow adsorption 6-well plate	MIN6 and HUVEC	Elucidate underlying mechanism of insulin secretion augmentation in the presence of HUVECs, investigation of beta-cell markers and, analysis of therapeutic potential in STZ-induced diabetic mouse	[284]
Pseudoislet	Agarose mold	βTC3 and RHMVEC	Spheroid characterization and endothelial sprouting in fibrin hydrogel to facilitate islet revascularization and function	[285]
Pseudoislet	Magnetic levitation	EndoC-βH3 and HUVEC	Effects of beta-cell and endothelial cell distribution and cellular interactions on beta-cell activity	[286]
Pseudoislet Spheroid	Hanging drop	MIN6 and hepatic/pancreatic stellate cell	Investigation of heterospheroid culture parameters on beta-cells, genetic engineering and testing stellate cells to express CCL22 to attract Tregs in vivo	[287]
Pseudoislet Spheroid	Non-adherent culture dishes followed by rotary cell culture reactor with high aspect ratio vessel (HARV)	MIN6 and PANC-1, PaTu-8988t, FA-6	Beta-cell and pancreatic cancer cell interaction	[288]
Pseudoislet Spheroid Organoid	AggreWell 400 24-well plate	Dissociated rat islet, HUVEC and hAEC	In vitro characterization, xenogeneic transplantation to facilitate vascularization and engraftment	[289]
Aggregate	Ultra-low attachment 60 mm diameter dish	Dissociated rat islet	Comparison of natural islet and reaggregated islet response against oxidative stress and function encapsulated within alginate microcapsule and beta-cell replacement in STZ-induced diabetic mouse	[290]
Aggregate	Culture medium dependent differentiation and aggregation	Human dental pulp stem cell	As a source for autologous stem cell therapy in diabetes	[291]
Aggregate	Culture medium dependent differentiation and aggregation	Human adipose tissue derived stem cell	As a source for autologous stem cell therapy in STZ-induced diabetic mouse	[292]
Aggregate	PEG microwell followed by 2 h on orbital shaker and culture under static cultures	MIN6	Defining optimum size with functional beta-cell mass	[293]
Aggregate	Ultra-low attachment 24-well plate on 3D rotator	MIN6	Mechanism of superior functional maintenance in 3D configuration	[294]
Aggregate	Non-adherent agarose microwell	Dissociated human islets and commercial murine cell lines	Optimization of spheroid properties to minimize mass transport limitation and, validation in vivo	[295]
Aggregate	6-well plate on a gyratory shaker	αTC-6 and βTC-1	Mimic heterogeneity and cellular communication	[296]
Aggregate	Hanging drop	Mouse sertoli cell and mouse dissociated islet cell	Development of cell-based immunosuppressive therapy in beta-cell replacement in STZ-induced diabetic mouse	[297]
Aggregate	96-well culture plate with V-bottomed wells	INS-1 and gelatin hydrogel microspheres	Effects of microspheres on beta-cell viability and functionality	[298]
Aggregate	Non-adherent agarose microwell chip	MIN6-B1 and HUVEC	Study of beta-cell function within membrane-based encapsulation devices and effect of endothelial cells	[299]
Aggregate	Gelatin–PLGA porous microwell scaffolds	RIN-5F	Effects of dimensionality and microwell size on aggregation and function of beta cells	[300]
Cluster	12-well microplate	Dissociated rat islet cells transfected to express exendin-4	Investigation of a novel strategy for diabetes treatment with aggregates of genetically engineered islet cells, PEG-lipid coating and anti-CD154 mAb and tacrolimus supplementation	[301]
Cluster	Culture medium dependent differentiation and aggregation	Human adipose tissue derived stem cell	Characterization of a beta-cell source in stem cell therapy in diabetes	[302]
Cluster	Hanging drop	Dissociated rat islet	Potential to be an alternative for native islets and superiority upon transplantation with matrigel in STZ-induced diabetic mouse	[303]
Cluster	Culture medium dependent differentiation and aggregation in 6-well plates on an orbital shaker	Human pluripotent stem cell	As a source for stem cell therapy with dynamic response and in diabetes in vivo	[304]
Cluster	Microwell bag	hiPSC line (1231A3 and Ff-I14s04) derived insulin secreting cell	Advancement of scalability, sterility and operability	[305]
Cluster	AggreWell 400/800 microwells as a part of differentiation protocol	Human pluripotent stem cell	Improvement of stem-cell derived beta-cell establishment protocol for diverse applications such as small molecule screening	[306]
Cluster Spheroid	Hanging drop	Dissociated rat islet and human bone marrow MSC	Capacity of MSCs to benefit in islet transplantation outcome by angiogenesis and anti-apoptosis	[307]
Cluster Spheroid	Hanging drop	Dissociated rat islet and curcumin-loaded PLGA microspheres	Ability of beta-cell clusters to overcome hypoxia-induced death of transplantation through curcumin	[308]
Vascularized islet	Low cell-adhesion 96-well plate with U-bottomed conical wells	Islet, HUVEC and human MSC	Development of vascularized islet organoid transplants to improve therapeutic efficacy	[309]
Islet-like structure	During culture medium dependent differentiation (incubation with protamine chloride)	Human liver stem-like cell	Endocrine differentiation into islet-like structures and beta-cell replacement potential in STZ-induced diabetic mouse	[310]
SC-islet	As a part of culture medium dependent differentiation (6-well AggreWell 400 plates followed by ultra-low attachment plates)	Human H1	Detailed characterization from cytoarchitecture to insulin secretory machinery	[311]
Stem-cell derived islet	AggreWell 400 plates and vertical-wheel bioreactors as a part of differentiation protocol	hiPSC	Generation and economical optimization of islet-like cluster with bioreactor included system and, characterization as well as analysis of effective islet transplantation mass	[312]
Islet microtissue	96-well hanging drop system	Dissociated human islet	As a model to determine the protective capacity of liraglutide on GSIS, cytokine secretion and T cell infiltration in islet microtissue-PBMC co-cultures	[313]

**Fig. 2:**
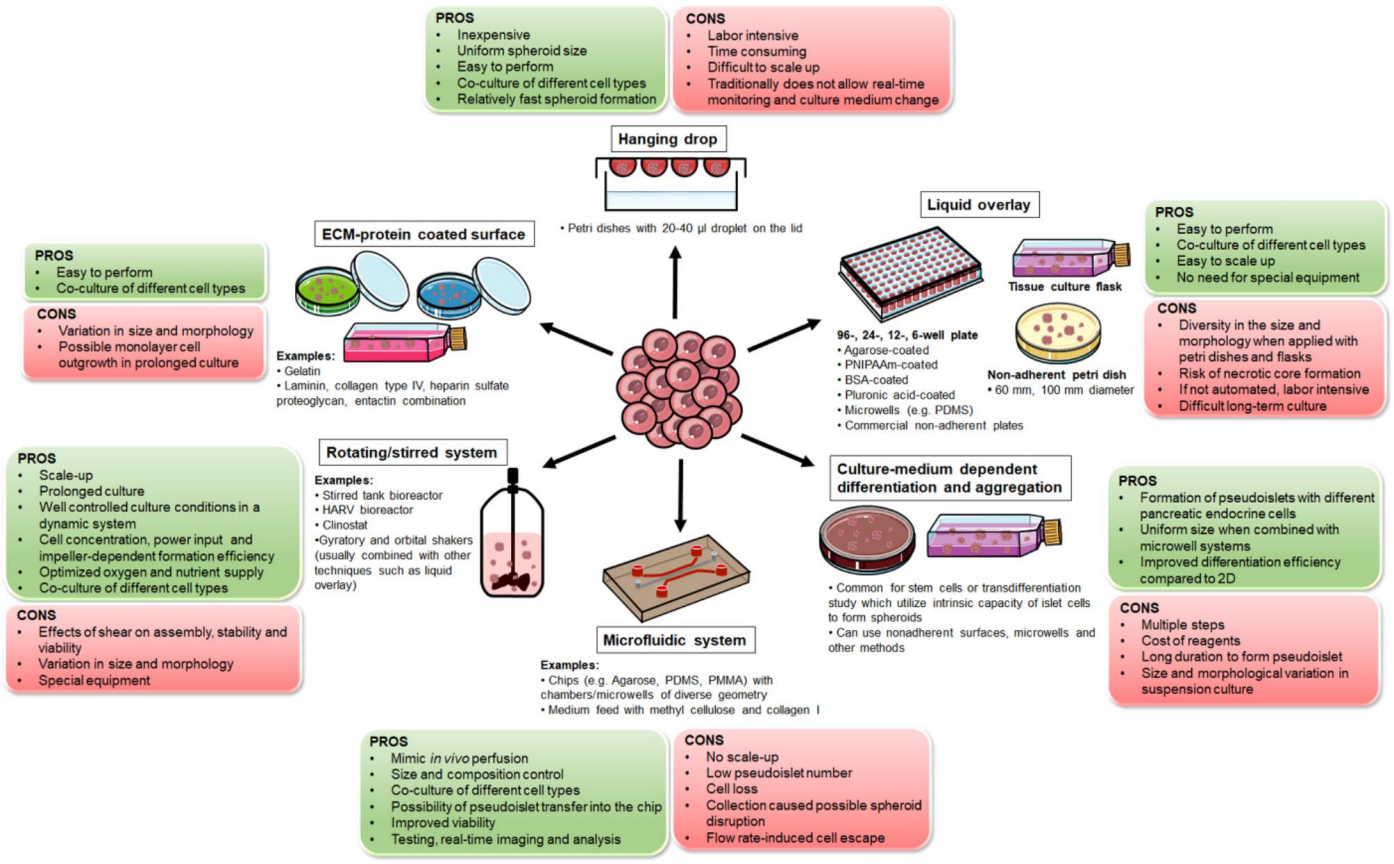
The most common pseudoislet assembly techniques. Although less widely applied, techniques such as magnetic levitation, assembly through layer-by-layer coated single cells, encapsulation and bioprinted molds are also reported (not shown in the figure). Figure 2 contains images from Servier Medical Art, which were modified in color, shape and size where necessary. Servier Medical Art (smart.servier.com) is licensed under a Creative Commons Attribution 4.0 International License (CC BY 4.0) (https://creativecommons.org/licenses/by/4.0/)

In general, artificial islets are produced from single beta-cells or with the combinations of the endocrine and non-endocrine cells of the pancreas. In literature, spheroid [249], islet spheroid [237], mixed (multicellular) spheroid [241, 248], spheroid cluster [246], homospheroid and heterospheroid [250], organoid [260], pseudoislet [274], SC-islet (organoid) [262], engineered pseudoislets [285], heterotypic pseudoislet [286], islet-like cell aggregate [292], aggregate [298], composite aggregate [299], coaggregate [297], cluster [306], islet cell cluster [303] and islet-like cluster [305] are common terminologies for the assemblies of islet-like 3D structures. In Table 1, for simplicity, spheroid, pseudoislet, cluster and aggregate terms are used, and for the terms which do not fit these classification, the nomenclature in the original paper has been used such as vascularized islet [309], islet-like structure [310], SC-islet [311], stem-cell derived islet [312] and islet microtissue [313]. Nevertheless, resulting 3D systems are fundamental for T1D research and clinics. In this review, they will collectively be referred to as pseudoislets for further simplification. It is noteworthy to mention that scaffold-free systems in the diabetes are not limited with the pseudoislet systems, but also cell-sheet engineering is another branch of this technology [314–316], yet here, focus will be on the pseudoislet systems. Various techniques are reported for the formation of pseudoislets (Table 1**and** Fig. 2). Although each has unique benefits and drawbacks [233, 250–252, 255, 263, 270, 278, 288, 302, 304, 311, 317–320], efficient formation of the pseudoislets is routinely performed for the diabetes research worldwide.

### Applications of artificial Islets

Islet transplantation has the capacity to extend the number of treated diabetic patients, but inadequacy of cell source still limits its widespread application as cell-based therapy. One of the factors involved in the insulin-free treatment is 3D configuration which influences stimulation and secretion of insulin and islet viability, as cell-cell interactions regulate diverse beta-cell aspects including phenotype maintenance, insulin expression and secretion, and viability. As a result, 3D culture systems are increasingly employed in beta-cell replacement therapy in vitro and in vivo [238, 251, 294, 317, 321]. In literature, mainly combination of transformed cells has been studied for the development of pseudoislets (Table 1) which are intended for research rather than clinics. In research, pseudoislet strategy controls the cell number to reach the euglycemia [268] and prevents unlimited growth after transplantation [235, 238] which are essential in the clinical applications. Ability to be produced in the large scale up to 1 L [255] and incorporation of not only insulin secreting cells, but also other endocrine cells which secrete glucagon and somatostatin [239] is achievable with the pseudoislets. The islet size in the resulting beta-cell culture is another indicator to achieve optimum viable pseudoislets with the endocrine response. Established pseudoislet formation techniques can also result in hypoxia-induced cell death in especially large pseudoislets [268]. However, it can be controlled in the pseudoislets, thus hypoxia-induced death can be minimized [303].

**Fig. 3:**
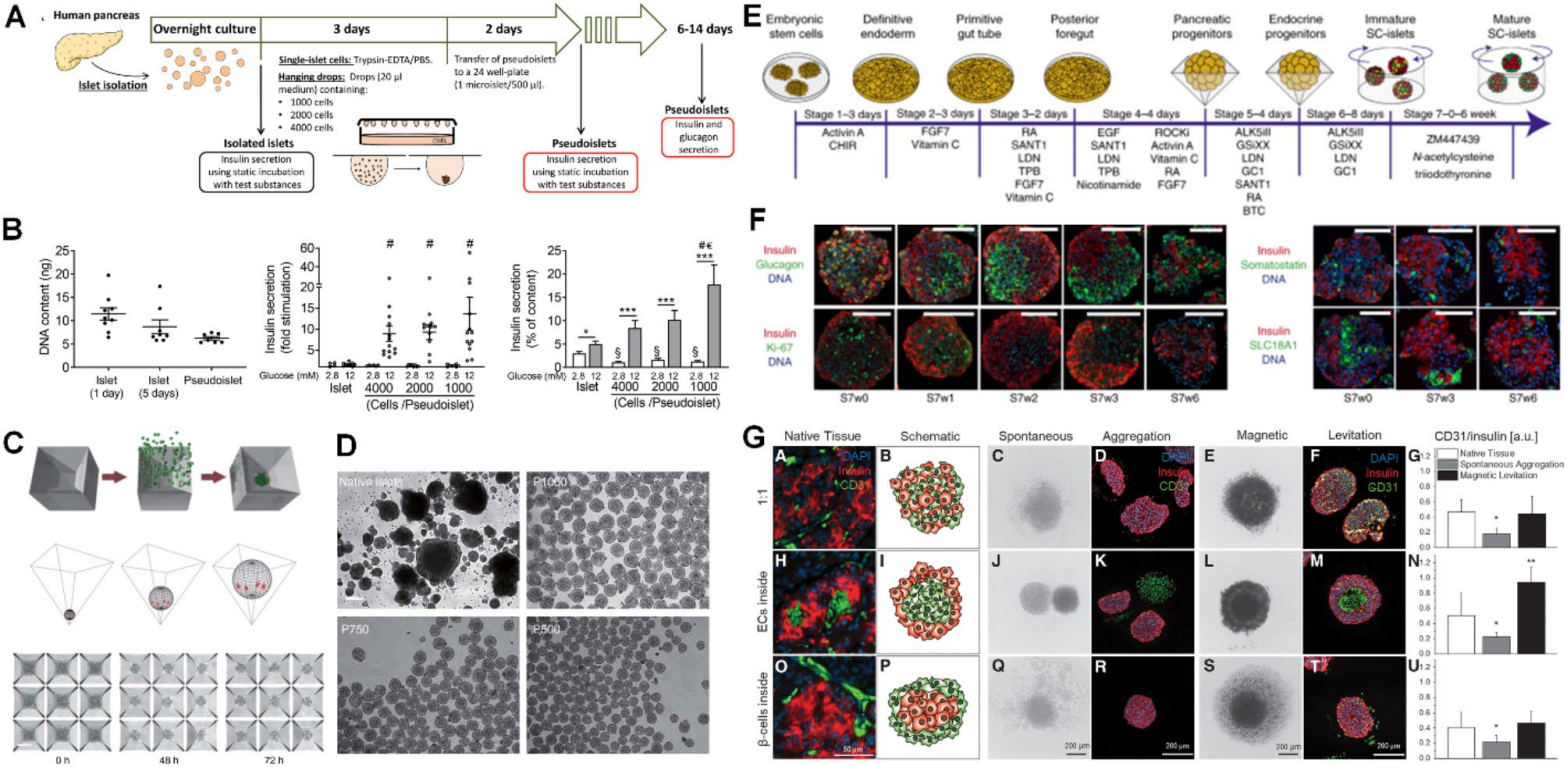
Examples of several methods to form pseudoislets and contributions to artificial islet research. **(A)** Design of human pseudoislets by hanging drop technique with different initial cell numbers. **(B)** (Left) Comparison of DNA content of native islets and human pseudoislets (cultured for 2 days and initial cell number: 2000 cells). (Middle) Fold stimulation of native islets and human pseudoislets in GSIS. (Right) Insulin secretion (% of insulin content) of native islets and human pseudoislets in 2.8 mM and 12 mM glucose. **(C)** Human pseudoislets formed via centrifugation of a suspension of single cells in square-pyramidal microwells for 0–72 h. **(D)** Controlled, consistent pseudoislet size in square-pyramidal wells with initial cell numbers of 1000, 750 and 500 human islet cells per well. Scale bars: 200 μm. **(E)** Differentiation protocol for SC-islets from human embryonic stem cell line, H1. **(F)** Immunostaining of SC-islets obtained via protocol in (E) for insulin, glucagon, somatostatin and SLC18A1, and DNA staining at stage 7 culture. Scale bars: 100 μm. **(G)** (Left) Brightfield and immunofluorescence images. (Right) Quantification of CD31^+^ cells of native human islets, pseudoislets (EndoC-βH3 cells and HUVECs) by spontaneous aggregation and magnetic levitation. Scale bars: 50 μm for native tissue and 200 μm for pseudoislets. GSIS: Glucose stimulated insulin secretion, HUVEC: Human umbilical vein endothelial cells. **(A)** and **(B)** are reprinted from Lorza-Gil et al. [274] published by Springer Nature and are licensed under Creative Commons Attribution 4.0 International License (http://creativecommons.org/licenses/by/4.0/). Labels (A, B, C, D) of original article are removed. **(C)** and **(D)** are reprinted from Yu et al. [272] published Springer Nature and are licensed under Creative Commons Attribution 4.0 International License (http://creativecommons.org/licenses/by/4.0/). Labels (a, b, c, d) of original article are removed. **(E)** and **(F)** are reprinted from Balboa et al. [311] published by Springer Nature and are licensed under Creative Commons Attribution 4.0 International License (http://creativecommons.org/licenses/by/4.0/). Labels (a, b) of original article are removed. **(G)** is reprinted from Urbanczyk et al. [286] published by Mary Ann Liebert, Inc. and is licensed under *Creative Commons CC BY* (http://creativecommons.org/licenses/by/4.0/)

A unique feature of these pseudoislets is their ability to resemble native islet organization. Walker et al. reported that reaggregated human islet cells maintain cellular identity, as the beta-cell (PDX1, NKX6.1) and the alpha cell transcription factor markers (MAFB, ARX) are preserved. Pseudoislet proliferation, apoptosis, architecture and dynamic insulin secretion profile were analogous to the native human islets which made them ideal candidates, for example, to investigate Gi and Gq receptors on the alpha- and beta-cell activities [277]. Also, human islet cell pseudoislets formed via hanging drop (Fig. 3A) reflect the uniform size of pseudoislets in contrast to human islets, as DNA content distribution is in narrow range and improved responsiveness to glucose can be achieved compared to native islets (8–13-fold stimulation vs. 2-fold stimulation) (Fig. 3B) [274]. Alternatively, microwells with specific geometry can allow spherical assembly of beta-cells with uniform pseudoislet sizes in contrast to native islets (Fig. 3C-D) [272]. Additionally, rearrangements as 3D pseudoislets successfully strengthen the physiological processes in the cells such as human clonal EndoC-βH1 and EndoC-βH5 cells, and overexpression of gap junction units of connexin 36 in the rodent INS-1 832/13 cells positively alters stimulation index [321].

Although pseudoislets can be obtained with similar endocrine cell ratios, there is a possibility that the distribution of the endocrine cells is not adjusted accordingly, and the basal insulin levels and insulin secretion are strong in contrast to the native islets. Still, they have a potential to normalize the glucose levels in the STZ-induced diabetic mice [275]. However, donor-based changes in GSIS and mainly higher insulin secretion are reported when human islet-cell based pseudoislets were treated with stimulators such as palmitate and forskolin at 12 mM glucose. In some cases, lack of endocrine cells and exocrine cells can be another deviation from native human islets [274]. The proinflammatory cytokine gene (IL1B, CCL2, CXCL8) and the ECM marker (ASPN, COL1A1, COL4A1) expressions are other altered parameters compared to the native islets [271], and loss of endocrine activity and display of the pancreatic duct characteristics in the culture were reported [322]. Even with such differences, pseudoislets are central to diabetes research and possibly to clinical transplantation. They, for instance, show localization in the target organ, can control blood glucose levels and are positive for insulin in the transplants [323]. As multicellular mimics of the islets, they can be used to establish main hallmarks in the T1D development. As in vitro tools in the cytokine mediated injury, in the activated peripheral blood mononuclear cell and HLA-A2-restricted preproinsulin-specific cytotoxic T lymphocyte attack, they can be manipulated to design separate conditions for each case, and the individual or combinatorial destructive activity on the beta-cell insulin release and the survival can be analyzed. This platform enables testing compounds such as liraglutide, gliclazide and exendin-4 to fight against the inflammatory attack [313]. Moreover, they can be studied to assess the effects of lysophosphatidylcholines on the Gpr40, Gpr55, and Gpr119 receptor stimulation and the insulin release [276], as well as be transferred to the microfluidic devices to establish a pancreas-on-chip for the disease modeling and drug screening [253]. Their applications to analyze the pancreatic cancer cell-beta-cell communication [288], as model beta-cell systems for the insulin detection system development [262], to design nanoencapsulation by the layer-by-layer assembly [264] or to assemble the pseudoislets by the layer-by-layer ECM protein coating [243] were recorded.

Equally, the source for the beta-cells is also a critical factor for the beta-cell replacement therapy in T1D. As donation is limited and for the research, continuous isolation is needed, other options as beta-cell sources are investigated. So far, reprogrammed islet alpha- and gamma-cells [273], pancreatic exocrine cells [324], transdifferentiated human liver cells [251], stem cells such as dental pulp stem cells [291], adipose-tissue derived stem cells [292], human pluripotent stem cells [306], omentum derived stem cells [256], liver stem-like cells [310] and human umbilical cord mesenchymal stem cells (MSC) [325] were tested as the (patient-specific) cell-based therapeutics. To direct the cellular fate, differentiation protocols are established by combining, for example, adherent culture, chemical treatment and induction of aggregate formation (Fig. 3E). By this way, for example, embryonic stem cells-derived pseudoislets with glucagon, somatostatin and insulin positive cells can be assembled and up to 6 weeks, proportion of insulin-positive monohormonal cells can be retained (~ 40%) (Fig. 3F) [311]. In one of the recent studies, the differentiated cells form final clusters with sizes of 172 ± 34 μm in diameter in suspension culture showing consistent sizes in contrast to islets and are positive for dithizone (DTZ) stain (zinc-chelating dye for beta-cell staining). Although these pseudoislets are positive for beta-cell markers (C-peptide, PDX1 and NKX6-1) indicating the beta-cell fate, small amount of glucagon positive and polyhormonal cells were recorded. Challenge of these pseudoislet cells in static and dynamic GSIS showed that simulation index (3.0 vs. 3.2) and dynamic insulin secretion profiles are similar, but lower insulin release was evident in different phases of release pointing an important limitation [304]. Although EndoC-βH1 pseudoislets are similar to islet assembly and this organization improved stimulation index (4.5 vs. 2.6 in monolayer culture), their level of insulin secretion awaits for improvement (mouse islet stimulation index of 16.4) [321].

The ability of the cells to interact with each other further opened the possibility to incorporate non-endocrine cells which are beneficial to reduce transplanted cell number, improve insulin secretion, contribute to ECM deposition and provide immunoprotection against cytokine attack. Among these, endothelial cells [241, 299], fibroblasts [241] and MSCs [250] have notable effects. In design of pseudoislets for the beta-cell replacement therapy, beta-cell heterogeneity in the pancreas is a useful strategy to obtain useful transplants, as FAC-sorting of the beta-cells based on CD63 expression showed that CD63^high^ beta-cells secrete more insulin, and alternative manipulation might be required to enhance the functionality of the other subsets [283]. As these systems are required to be scaled-up, several factors such as metabolic alterations, growth kinetics, cell seeding density, yield and stirrer types should be considered [255, 305, 326]. Further, commercial beta-cells used for the formation of pseudoislets are derived from for instance, mouse insulinoma and they display differences in insulin expression and content, as well as can show features that belong to both alpha-cells and beta-cells [249]. Other drawbacks include functional impairment at high passages [327], diversity in the amount of secreted insulin between cell types [276] and beta-cell dedifferentiation due to continuous stimulation which restrict the use of cells such as MIN6 cells [328].

Although life-long immunosuppression is another aspect of organ/cell transplantation to prevent rejection, it has adverse effects even on the beta-cells transplanted. Replacement of these drugs in the endocrine tissue engineering is suggested to be mainly with the development of the immunoisolation barriers [270, 329]. Additionally, a cell-based immunosuppressive regimen is proven to be useful to eliminate the use of the drugs and, consequently, can still protect the cells against immune reaction in the alloimmunity and even against the autoimmunity. One group of such cells are Treg cells which have the capacity to suppress the proliferation and activation of diverse immune system cells such as lymphocytes, NK cells and antigen-presenting cells. To support this strategy, Takemoto et al. transplanted pseudoislets of Tregs and islet cells intraportally to the STZ-induced diabetic mice and observed that this approach held a potential to reach the normoglycemia and that some insulin positive cells were retained in the animals after 120 days whereas massive destruction was evident in the pseudoislets without Tregs. This study points out the conclusion that Treg can provide immunoprotection to the transplanted islets to some extent [329]. Similarly, the transplantation of the pseudoislets composed of Sertoli cells and islet cells yielded encouraging outcomes, since Sertoli cells act as barriers to immune cell migration and Sertoli cell-derived factors contribute to shape the immunoprivileged testis environment as the unique cues for the immunoprotection of the cell transplants [297]. It is also possible to genetically engineer cells such as stellate cells to express Treg recruiting factor of C-C motif chemokine ligand 22 (CCL22). Incorporation of these cells into the pseudoislets can attract more Tregs to the area of graft, therefore, it has a potential to support the survival and capacity of the transplant to reach proper blood glucose and insulin levels [287].

Oxygen levels positively regulates the insulin secretion, thus, hypoxia adversely affects GSIS [279]. This effect is also valid in the developing and prolonged culture of the pseudoislets, as hypoxia will adversely affect the existence and the function [330]. For this purpose, a system that controls the oxygen transfer has utmost importance. For instance, Lee et al. designed a polydimethylsiloxane (PDMS) based microwell array with 10–1050 μm film thickness to manipulate the oxygen transfer and were able to demonstrate the effect of more oxygenated system on the insulin secretion and survival [244]. Incorporation of the microspheres to provide a route for the oxygen and nutrient transfer [298], or the microspheres loaded with drugs such as curcumin as anti-apoptotic agents and for the increase of the insulin release [308], pseudoislets with MSCs to fight the hypoxia by the soluble signals and the angiogenesis [307], human amniotic epithelial cells to resist the hypoxia and sustain the endocrine cell response [258] thereby, can serve for the desired outcome. Alternatively, size of the pseudoislets can be controlled with specifically designed microwells so that possibility of cell death and hypoxic core formation decreases. In one such studies, Ichihara et al. fabricated agarose-based microwells and rat-islet cell pseudoislets showed high level of cell death and development of hypoxia in larger pseudoislets (formed in 500 μm diameter wells) whereas in smaller ones, cell death was minimal and even lower than native islets [268].

Oxidative stress for the islets is also associated with the loss of the vessels during the isolation and with the lack of vascularization initially after transplantation. These processes have unfavorable impact on the islets, thus consequences of the transplantation and number of the transplants per patient. Therefore, supplementation of the transplants with compounds reducing the oxidative stress is beneficial for the patients. In the discovery of such compounds, pseudoislets assembled with the alpha- and beta-cells in the low attachment microplates provide example data. Sulfisoxazole, hydroxyzine dihydrochloride and tribenoside are some compounds reported by using pseudoislets as screening platforms [282]. Instead, vascularization-based guidance to reduce the effects of oxidative stress is also applicable. Although islets are 3D cell assemblies that significantly model beta-cell contacts, modulate calcium signaling and maintain ECM proteins, similarly vascular cells support beta-cells and are natural components in the pancreas [266]. Islets in the pancreas are typically highly vascularized to assist the supply of the metabolic requirements of the cells as well as for the endocrine function. They occupy less than 1% of pancreas mass, but they receive 15–20% of the pancreatic blood flow. Within such an organization, endothelial cells and beta-cells interact, secrete basement membrane ECM and, endothelial cells are important to export the insulin into the blood circulation. Thus, loss of beta-cell transplants due to the lack of vascularization becomes inevitable [285, 286]. Considering the organization in the pancreas, a 3D system of beta-cells and endothelial cells can be superior over beta-cell monolayer culture in the insulin secretion and ECM synthesis (collagen-IV, laminin), and further viability can be sustained [266]. A co-culture of these two types of cells as stable pseudoislets under controlled conditions can be a concept to reduce the impact of the multiple transplantation and can be unlimited source in the disease study. In the human pancreas, endothelial cell and beta-cell localization occur as heterogenous distribution (i.e. beta-cells covered by endothelial cells and, vice versa of the latter). These unique patterns are probable indicators which alter GSIS and eventually transplant success. As a result, research comparing these distributions and cell-cell interactions with human beta-cells offers a unique opportunity to design an ideal beta-cell response in the transplants. For example, a study by Urbanczyk et al. mimics three possible localization conditions created by magnetic levitation allowing desired cell distribution with improved endothelial cell incorporation (Fig. 3G) and, derived stable pseudoislets revealed that all three conditions have similar cell death, yet beta-cells covered by endothelial cells present higher insulin secretion and E-cadherin (a stimulator for the insulin expression, used in the cell-cell interactions of beta-cells) expression [286]. As pseudoislets with endothelial cell components can facilitate beta-cell engraftment, research on beta-cell and endothelial cell pseudoislets in a biomaterial such as fibrin [285] or incorporation of microvascular fragments can rescue beta-cell from hypoxia [260]. As third component, amniotic epithelial cells can be combined to regulate endothelial cell-based vascularization by angiogenic factors such as VEGF-A and to remodel ECM with MMPs. Additionally, these cells influence islet-cell PDX-1 and GLP1R expression by EGF which eventually contributes to the glycolytic gene and VEGF-A secretion improving not only beta-cell survival and insulin metabolism, but also vascularization [289].

After formation, pseudoislets are generally studied in static cultures, but a dynamic culture with a flow system might provide unique differences in the pseudoislet transcriptomics and metabolomics by simulating the flow experienced in the body. In one of such studies, Essaouiba et al. demonstrated that 3D biochips containing human induced pluripotent stem cell (hiPSC) derived beta-cell pseudoislets increased, for instance, hypoxia-inducible factor activity, glucose metabolism and enriched insulin as well as glucagon pathways, activated SOX17 and MAFB motifs for the maintenance of the differentiated state of beta-cells and their insulin secretion [254]. On-chip systems are also advantageous for the control of the environment, nutrient delivery, pseudoislet loading, time-dependent analysis and imaging, as well as end-point studies [278]. In recent reports, this dynamic system is used as a platform to build pseudoislets [259], to test drug efficacy [115, 281] as well as to model the effects of the flow-induced mechanical stress, chronic glycemia and lipidemia on the beta-cell gene expression in the diabetes [245].

The quality of the islet population, loss of the islets after transplantation due to lack of vascularization and post-transplantation immune activity restrict the patients receiving the islet transplants. Genetic manipulation of the islets is one strategy to overcome these barriers, as angiogenetic factor expression, control of the insulin production and transplant-mediated suppression of immune system are encouraging. This route involves efficient transfection of single cells which are reassembled into the pseudoislets during gene delivery. For instance, Voznesenskaya et al. reached the conclusion that transduction with AAV8 during pseudoislet formation yields 2.5-fold higher expression than intact isolated islets with improved transfer to the pseudoislet core [261]. Van Krieken et al. transduced the islet cells during pseudoislet aggregation by adenovirus carrying V1b receptor gene that can initiate GSIS by phospholipase C pathway and altering Ca^2+^ levels. [Arg^8^]-vasopressin induced beta-cell activity in transduced pseudoislets and supported insulin secretion in vitro [275]. Genetic engineering of islet cells to express exendin-4 (a GLP-1 analogue) to increase the insulin secretion and decrease the apoptosis [301], and poly(ethylenimine) (PEI)-mediated IL-10 gene delivery before aggregation [237] can protect the beta-cells and advance the insulin secretion.

In addition to applications in vitro, islets and islet-like tissues have been in clinical trials for the last couple decades. In clinical trials database (https://clinicaltrials.gov/), search on “Type 1 Diabetes” and “Islet Graft” shows 33 different studies and “Type 1 Diabetes” and “islet transplantation” as keywords show 142 studies. “Type 1 Diabetes” and “Cell Therapy” results in 151 results. Among the studies mentioned, in addition to islet transplantation, stem cells are the major focus due to their immunomodulatory and differentiation potential, as well as due to stem-cell derived factors for beta-cell regeneration. Stem cells can be used in as a part of cell therapy derived from allogenic sources to protect beta-cells (NCT04061746), to manage C-peptide levels (NCT01121029), to educate immune system cells (NCT02624804, NCT04011020, NCT01996228, NCT01350219), to determine safety and/or efficiency in T1D patients (NCT01143168, NCT00807651, NCT00703599, NCT05308836), to regenerate beta-cells and even for re-differentiation into local tissues (NCT01374854). There are also trials on allogeneic stem cell-derived, insulin-producing islet cell therapy (NCT04786262) and encapsulated allogeneic stem cell-derived, insulin-producing islet therapy (NCT05791201).

Another group includes hiPSCs and MSCs where these cell classes are investigated as sources of islet-like tissues. In these reports, pilot/clinical studies showed glycemic control within 3–4 months showing the promise of this technology [331, 332].

## Conclusions and future directions

As T1D is increasingly diagnosed worldwide, development of new treatments is of interest for current efforts. Insulin treatment is the leading strategy in the clinics followed by the beta-cell replacement therapy to achieve glucose homeostasis, both of which are associated with serious drawbacks. Recent technologies enabled researchers to design scaffold-free artificial islets as endocrine models for purposes ranging from drug screening to pancreas physiology. A practical application is the utilization of so-called pseudoislets in beta-cell replacement therapy instead of exogenous insulin supply. These new systems are directed to address the challenges such as native organization, immunoprotection and functional improvements and, the results reported are more realistic compared to 2D monolayer culture and single cell containing transplants. Although various artificial islet models and their potential applications are established so far, the field remains to be in the preclinical stage due to factors such as standardization, beta-cell heterogeneity, requirement of complete mimic of cellular and acellular environment, donor shortage and lack of sufficient clinical trials. Thus, future research faces multiple challenges to translate the islet mimics into the clinics. Moreover, today, the majority of the pseudoislet research is shaped around incorporation of beta-cells and other endocrine cell types as well as endothelial cells with different techniques. Further, cells such as Tregs, Sertoli cells and stem cells to provide immunoprotection, and fibroblasts and stem cells to improve insulin secretion are added and efficiently islet-look alike and function-like cell assemblies are produced. However, cell-cell contact, and cell-soluble factor mediated control of beta-cell metabolic capacity are not limited to only these types of cells. In the future, with more genetically stable cells, contribution of nervous system to pseudoislet electrophysiology should be analyzed and flow-based systems rather than assay based dynamic perifusion systems should consistently be used to fully elucidate the promise of these 3D assemblies. An important point will also be addressing the issue of autologous islet-derived systems for personalized medicine.

## Data Availability

No datasets were generated or analysed during the current study.

## Abbreviations

ANG: Angiopoietin
CAF: Cancer-associated fibroblast
CCL22: C-C motif chemokine ligand 22
CHPOA: Acrylate modified, cholesterol bearing pullulan
CXCL: C-X-C motif chemokine ligand
CXCR2: CXC chemokine receptor 2
Dlg: Discs large protein
DTZ: Dithizone
ECM: Extracellular matrix
EGF: Epidermal growth factor
ESAM: Endothelial cell selective adhesion molecule
GLP-1: Glucagon-like peptide-1
GLP1R: Glucagon-like peptide-1 receptor
GLUT: Glucose transporter
GPCR: G-protein coupled receptor
GSIS: Glucose stimulated insulin secretion
HGF: Hepatocyte growth factor
hiPSC: Human induced pluripotent stem cell
HLA: Human leukocyte antigen
HUVEC: Human umbilical vein endothelial cell
ICAM-1: Intercellular adhesion molecule-1
IFN: Interferon
IGF: Insulin-like growth factor
IL: Interleukin
JAM: Junctional adhesion molecule
LIF: Leukaemia inhibitory factor
MBP-FGF2: Maltose-binding protein-basic fibroblast growth factor 2
MMP: Matrix metalloproteinase
MSC: Mesenchymal stem cell
N-CAM: Neural cell adhesion molecule
NO: Nitric oxide
P2X7R: P2X purinoceptor 7
Par3: Partitioning defective 3 homologue
PD-1: Programmed cell death protein-1
PDGF: Platelet-derived growth factor
PDGFRβ: Platelet-derived growth factor receptor-β
PD-L1: Programmed death-ligand 1
PDMS: Polydimethylsiloxane
PECAM-1: Platelet endothelial cell adhesion molecule-1
PEG: Poly(ethylene glycol)
PEI: Poly(ethyleneimine)
PMMA: Poly(methyl methacrylate)
PNIPAAm: Poly(N-isopropylacrylamide)
PSC: Pancreatic stellate cell
PTPN22: Protein tyrosine phosphatase non-receptor type 22
RIM2: Rab3-interacting protein
STZ: Streptozotocin
T1D: Type 1 diabetes
T2D: Type 2 diabetes
TGF: Transforming growth factor
TGF-βR: Transforming growth factor β receptor
TNF: Tumor necrosis factor
VDCC: Voltage-dependent calcium channel
VE-cadherin: Vascular endothelial cadherin
VEGF-A: Vascular endothelial growth factor A
VEGFR: Vascular endothelial growth factor receptor
ZO: Zona occludens
